# Optimization and Prediction of Ibuprofen Release from 3D DLP Printlets Using Artificial Neural Networks

**DOI:** 10.3390/pharmaceutics11100544

**Published:** 2019-10-18

**Authors:** Marijana Madzarevic, Djordje Medarevic, Aleksandra Vulovic, Tijana Sustersic, Jelena Djuris, Nenad Filipovic, Svetlana Ibric

**Affiliations:** 1 Department of Pharmaceutical Technology and Cosmetology, Faculty of Pharmacy, University of Belgrade, 450 Vojvode Stepe Str., 11221 Belgrade, Serbia; djordje.medarevic@pharmacy.bg.ac.rs (D.M.); jelena.djuris@pharmacy.bg.ac.rs (J.D.); 2 Department for Applied Mechanics and Automatic Control, Faculty of Engineering, University of Kragujevac, 6, Sestre Janjic Str., 34000 Kragujevac, Serbia; aleksandra.vulovic@kg.ac.rs (A.V.); tijanas@kg.ac.rs (T.S.); fica@kg.ac.rs (N.F.); 3 Bioengineering Research and Development Center (BioIRC), 6 Prvoslava Stojanovica Str., 34000 Kragujevac, Serbia

**Keywords:** three-dimensional printing, additive manufacturing, digital light processing technology, printlets, neural networks, optimization, prediction

## Abstract

The aim of this work was to investigate effects of the formulation factors on tablet printability as well as to optimize and predict extended drug release from cross-linked polymeric ibuprofen printlets using an artificial neural network (ANN). Printlets were printed using digital light processing (DLP) technology from formulations containing polyethylene glycol diacrylate, polyethylene glycol, and water in concentrations according to D-optimal mixture design and 0.1% *w*/*w* riboflavin and 5% *w*/*w* ibuprofen. It was observed that with higher water content longer exposure time was required for successful printing. For understanding the effects of excipients and printing parameters on drug dissolution rate in DLP printlets two different neural networks were developed with using two commercially available softwares. After comparison of experimental and predicted values of in vitro dissolution at the corresponding time points for optimized formulation, the *R*^2^ experimental vs. predicted value was 0.9811 (neural network 1) and 0.9960 (neural network 2). According to difference f_1_ and similarity factor f_2_ (f_1_ = 14.30 and f_2_ = 52.15) neural network 1 with supervised multilayer perceptron, backpropagation algorithm, and linear activation function gave a similar dissolution profile to obtained experimental results, indicating that adequate ANN is able to set out an input–output relationship in DLP printing of pharmaceutics.

## 1. Introduction

Three-dimensional printing (3DP) is an additive manufacturing process that allows the fabrication of three-dimensional solid objects of virtually any shape from a 3D model file [1,2,3]. The basic mechanism for most types of 3D printing is the same (layer-by-layer production of 3D objects from digital designs) [4], but the difference lies in input materials and operating principles. There are several types of 3D printing technologies: fused deposition modeling (FDM)—based on extrusion [5], selective laser sintering (SLS)—based on powder bed fusion [6], stereolithography (SLA), and digital light processing technology (DLP)—based on photopolymerization of the resin and others [2]. SLA 3D printing was the first rapid prototyping method developed and perhaps the most popular due to its superior resolution and accuracy [7]. DLP is a “sister technology” to SLA as the only significant difference between these technologies is the light source used to cure the resin. SLA printers use lasers combined with galvanometers to cure the resin while in DLP 3D printers, the light source is a specially developed digital light projector screen. Due to the presence of this screen, DLP is generally considered to be faster and more efficient than SLA [8]. The main drawbacks of SLA and DLP technology are the limited number of photocrosslinkable polymers that are available for medical applications, and these materials are currently not on the generally recognized as safe (GRAS) list of excipients [9].

Research in the field of oral drug delivery using SLA and DLP is still very limited. Wang et al. who fabricated 4-aminosalicylic acid and paracetamol loaded printlets, showed no drug degradation during the 3D printing process. [7]. In the study by Martinez et al. percentage of water in the initial formulation was varied, showing that the crosslinking density is slightly modified as the water content increases (up to 30%) and this dilution with water did not seem to significantly affect the speed at which the drug was released [9]. Influence of geometry on the drug release profiles was investigated by Martinez et al. [10]. In the study by Kadry et al. theophylline, as a model drug, and two photoreactive polymers, polyethylene glycol diacrylate (PEGDA) and polyethylene glycol dimethacrylate (PEGDMA), were used. Polymer concentration was varied to produce sturdy printlets with minimum polymer concentration applying, for the first time, DLP technology [11]. Optimization techniques have not yet been applied in 3D DLP fabrication and optimization of solid oral dosage forms. 

The most frequently used optimization technique is design of experiments (DoE), but with the development of computer science, artificial neural network (ANN) have attracted a lot of attention. Despite the advantages of DoE-based polynomial model fitting, often the developed models show bad fit resulting to a poor optimum estimation. An alternative approach that has been successfully applied in cases where conventional DoE methods prove inadequate is the use of feed-forward ANNs [12]. Neural networks create their knowledge by detecting the patterns and relationships in data. It is a biologically inspired computer-based system formed from hundreds of single units, artificial neurons, connected with coefficients (units) which constitute the neural structure. The artificial neuron takes one or more inputs and creates an output, which is passed on to another neuron. One of the most useful advantages of artificial neural networks is their ability to generalize. The multilayered perceptron (MLP) neural network is one of the simplest ANNs and consists of an input layer, output layer, and one or more hidden layers of neurons. During the ‘training process’ the system is able to establish the relationship between inputs and outputs using algorithms designed to alter the weights of the connections in the network to produce a desired signal flow. Although MLP has proved efficient in solving an important number of pharmaceutical development problems, no single software or modeling algorithm can solve ‘all’ problems [13,14,15,16]. There are a few examples in literature describing combination of DoE and ANN with recognized possibility as a powerful tool in predicting optimal conditions from a low number of experiments [17]. DoE enable determination of the quantitative relationship between selected input variables and responses while ANNs often exhibit superior performance in prediction of the responses for given values of inputs [18]. ANN can be used in completing one portion of data in the experimental design data pool, resulting in satisfying results for some outputs, considering the number of experimental data used for modeling [19].

The aim of this work was to investigate the effects of formulation factors on printability as well as to optimize and predict extended drug release from cross-linked polymeric ibuprofen printlets using ANN created in two different softwares. For a meticulous investigation of the effects of excipients on drug release, ANN was used because it is highly recommended to present the complicated relations and strong nonlinearity between different parameters [20]. The prediction and optimization method was applied to the development of ibuprofen extended-release 3D printlets using MLP and the backpropagation algorithm with linear and log-sigmoid activation functions.

## 2. Materials and Methods 

PEGDA, average MW 700, was obtained from Sigma–Aldrich, Tokyo, Japan. Polyethylene glycol (PEG 400, average MW 400) was purchased from Fagron B.V., Rotterdam, The Netherlands. Ethanol, absolute was purchased from Honeywell Riedel-de-Haën™, Seelze, Germany and 2-propanol was obtained from Merck KGaA, Darmstadt, Germany. Ibuprofen (Ph. Eur. 9.0) was used as a model substance, PEGDA as the photopolymerizable monomer, while PEG 400 and water were used to alter the cross-linking density. PEG 400 is chemically similar to PEGDA and the difference is that does not have photopolymerizable terminal groups. Riboflavin (Ph. Eur. 9.0) was used as the photo-initiator. The photo-initiator converts to reactive radicals upon exposure to light to catalyze the polymerization of the formulation. In photopolymerization reactions different photo-initiators can be used, and riboflavin is reported as pharmacologically non-toxic photo-initiator [9,21,22]. 

### 2.1. Preparation of Photopolymer Solution

Based on preliminary experiments, lower and upper limits (% *w*/*w*) of each component were selected as follows: PEGDA (30.0–74.6%), PEG 400 (10.0–54.6%), water (10.0–30%), and amounts of ibuprofen and riboflavin were kept constant, 5.0% and 0.1% respectively. Eleven formulations were prepared according to D-optimal mixture design from Design Expert software 7.0.0 (Stat-Ease Inc., Minneapolis, MN, USA). Compositions of the formulations obtained by the software are given in Table 1. Firstly PEGDA, PEG 400, and ibuprofen were mixed with propeller mixer Heidolph RZR2020 (Heidolph, Schwabach, Germany) until complete dissolution (approximately 60 min). Riboflavin and water were added next, keeping the solution protected from light and with constant mixing until complete dissolution (approximately 45 min). Compositions of three test formulations were selected so that they differ from the previous 11 and were prepared in the same way. Approximately the same concentration of PEGDA and PEG 400 was selected in the placebo formulation. Percentage of water was varied from 10 to 30 in formulations F1–F11, based on which 15% of water was chosen in the placebo formulation. 

### 2.2. Printing Dosage Forms

**Figure 1:**
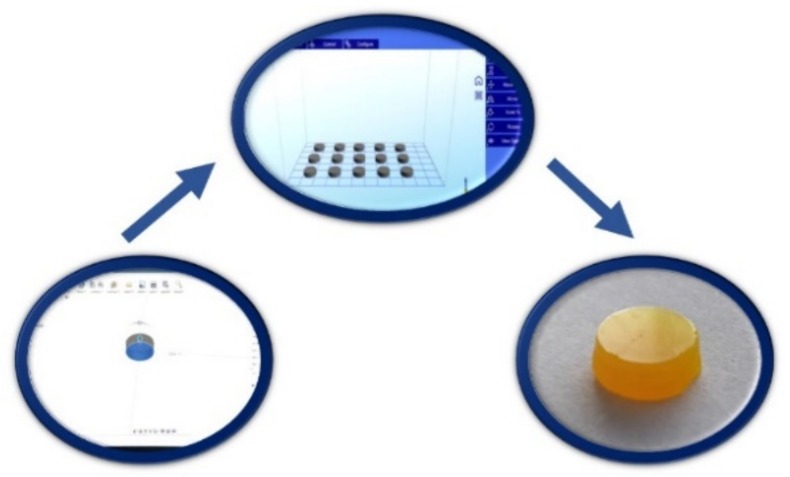
Digital light processing technology (DLP) printing process.

**Figure 2:**
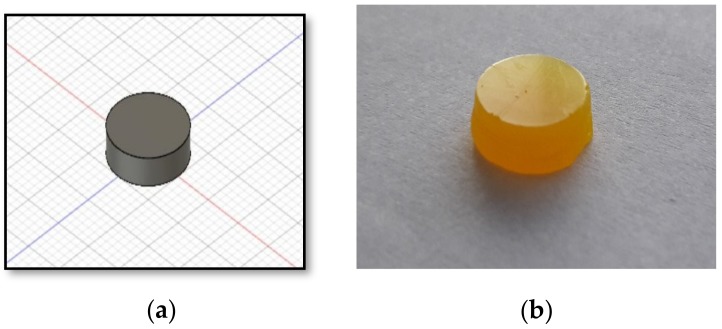
(**a**) 3D model of DLP printlet; (**b**) DLP printlet

In this study a DLP printer, based on photopolymerization process, was used for fabrication of solid oral dosage forms, called printlets. The DLP printer offers fast and efficient printing by projecting the light onto a whole layer at once, while the SLA printer prints each layer in a line by line pattern. The advantage of the Wanhao DLP printer is an open software and the possibility for adjustment of parameters for printing a particular mixture [11]. A schematic view of the printing process is shown in Figure 1. The template used to print the printlets (a cylinder, 10.00 mm diameter, 3.02 mm height) was designed with Autodesk Fusion 360 (Autodesk Inc, San Rafael, CA, USA) (Figure 2a) and exported as a stereolithography file (stl) into the 3D printer software (Creation Workshop X). All 3D printlets were printed with a Wanhao Duplicator 7 printer (Wanhao, Zhejiang, China) with layer thickness of 100 µm, bottom exposure 800 s, and 10 bottom layers. Trial-and error approach was used to establish exposure time for successful printing. In screening formulations, ibuprofen content was 5.0% and the water content was varied from 5.0% to 30.0%. The minimum exposure time which lead to solidification was selected. This criterion for exposure time allowed printing to be as short as possible. 

### 2.3. Characterization of Printlets

#### 2.3.1. Determination of Physical and Mechanical Properties

Three-D printed printlets were washed with 2-propanol to remove any uncured liquid formulation on the surface immediately after fabrication, then they were weighed and measured (diameter and thickness, *n* = 10) using a caliper. The breaking force of printlets (*n* = 10) was measured using a hardness tester Erweka TBH 125D (Erweka, Langen, Germany). Microscopic observations of placebo and optimal printlets were done under a polarized light microscope Olympus BX 51P (Olympus, Tokyo, Japan). Photos were acquired using cellSens Entry Version 1.14 software (Olympus, Tokyo, Japan).

#### 2.3.2. Determination of Drug Concentration in 3DP Printlets

Printed printlets were crushed using mortar and pestle (*n* = 3), and 200 mg of the crushed printlet was diluted with 10 mL of ethanol. Samples were placed in an ultrasonic bath Bandelin–Sonorex RK102H (Sonorex–Bandelin, Berlin, Germany) at room temperature and sonicated for 15 min to enhance extraction of ibuprofen. At the end of sonification, dispersions were cooled to room temperature and then filtered through a 0.45 µm Millipore filter (Merck Millipore Ltd. Carrigtwohill, County Cork, Ireland) A sample of 250 µL of the solution was diluted to 50 mL with phosphate buffer pH 6.8. Amount of drug in solution was determined using UV–Vis spectroscopy Evolution 300 (Thermo–Fisher Scientific, Waltham, MA, USA) at the wavelength of 221 nm. Corresponding placebo samples were analyzed in order to nullify the possible effect of other printlet constituents on drug absorbance.

#### 2.3.3. Dissolution Test Conditions

Drug release profiles were obtained using the paddle apparatus Erweka DT 600 (Erweka, Langen, Germany). The printlets were placed in 900 mL of phosphate buffer pH 6.8 for 8 h. The paddle speed of the USPII was fixed at 75 rpm, and the tests were conducted at 37 ± 0.5 °C. Buffer samples of 4 mL were withdrawn at predetermined time intervals, filtrated through a 0.45 µm Millipore filter (Merck Millipore Ltd. Carrigtwohill, County Cork, Ireland), and the absorbance of released ibuprofen was measured UV-spectrophotometrically at the wavelength of the relative maximum absorbance (221 nm). Studies were performed in triplicate.

#### 2.3.4. Kinetic Model

A number of mathematical models have been proposed to describe drug release from pharmaceutical delivery systems [23,24]. Drug release profiles were fitted into four mathematical models including zero-order, first-order, Higuchi, and Korsmeyer–Peppas.

#### 2.3.5. Differential Scanning Calorimetry (DSC)

DSC was used to study the thermal properties of placebo and optimal printlets. DSC analyses were performed on a DSC 1 differential scanning calorimeter Mettler Toledo AG (Analytical, Zurich, Switzerland). Accurately weighed 5–10 mg of samples (optimal and placebo formulation) were placed in pierced aluminum pans, and subjected to heating at 10 °C/min in the range of −50–200 °C under nitrogen purge gas flow of 50 mL/min. An empty pan was used as a reference.

### 2.4. Artificial Neural Network Modeling

To get better insight in an input–output relationship in DLP printing of ibuprofen printlets in ANN modeling, two artificial neural networks, using different commercially available software, were used. Each software has unique potential in solving problems.

(1) Neural Network 1. Commercially available STATISTICA 7.0 Neural Networks software (StatSoft Inc., Tulsa, OK, USA.) was used throughout the study. For prediction and optimization of ibuprofen release from 3D DLP printlets, supervised MLP and backpropagation algorithm with linear activation function were used. The data set was split into training (8 formulations), validation (2 formulations) and test (1 formulation) subsets. Amount of PEGDA, PEG 400, and water (% *w*/*w*) in formulations were selected as input factors affecting the release of ibuprofen. The cumulative percentage of ibuprofen release from 3D DLP printlets at time points of 1, 2, 4, 6, and 8 h was used as output data (Table S1). A trial and error approach, conducted by varying the number of layers and number of nodes in the hidden layer(s), was used to train the neural network. Learning rate and momentum were 0.6, the number of layers was varied from 3 to 10, and the number of nodes in the hidden layer(s) from 4 to 10. The criteria to choose the ˝best MLP model˝ were minimal test error and maximum coefficient of determination *R*^2^ for observed vs. predicted values. After the training process, the prediction ability of the developed network was examined by external validation with the unseen samples of three test formulations.
(2) Neural Network 2. Another approach was the usage of commercial software MATLAB R2014b (The MathWorks, Inc., Natick, MA, USA) to investigate the combination of process and formulation factors on optimization of ibuprofen release. A supervised MLP network and backpropagation algorithm with linear and log-sigmoid activation functions were used for the prediction. Percentage of PEGDA, PEG 400, and water in formulations were selected as input factors affecting the release of ibuprofen, as well as exposure times (s). The cumulative percentage of ibuprofen released after 2, 4, 6, and 8 h was the output data (Table S2). The most optimal MLP model was chosen based on the maximum R and minimal normalized mean square error between the calculated and target output for the test data. After the training process was finished, the prediction was examined by external validation with the unseen test (optimal formulation).

### 2.5. Optimization of 3D Printed Printlets

D-optimal mixture design was established by data predicted using evaluated MLP, because this approach of using DoE-based modeling to decipher the black-box nature of the ANNs resulted in satisfying results. Data obtained using DoE enable the development of more accurate models and improve process understanding [19]. The desirability function approach has been proven to be a useful statistical tool, the most widely used in industry, for solving multi-variable problems and optimization of one or several responses [19,23]. The objective function, D(X), called the desirability function is used in this method. It reflects the desirable ranges for each response (di) from zero to one (least to most desirable respectively). The simultaneous objective function is a geometric mean of all transformed responses. The numerical optimization technique was used to generate the composition of formulation with desirable drug release. The criteria for the selection of the optimal formulation were the percentage of in vitro release at time points of 2, 4, 6, and 8 h—not more than 30%, 60%, and 70%, and not <80%, respectively. The importance of the first two goals was set with two pluses, and the importance of the next two goals was set with three pluses, as they were more significant. After determination of the optimal composition of the formulation, the formulation was prepared, characterized by dissolution test, and the obtained results from the dissolution test were compared with the predictions by neural network 1 and neural network 2. Predictability was expressed through calculation of the coefficient of determination (*R*^2^), *f*_1_ (difference factor) and *f*_2_ (similarity factor). Difference and similarity factor are represented in Equations (1) and (2).

(1) $$f_{1}=\left(\frac{\sum_{t=1}^{n}\left|Rt-Tt\right|}{\sum_{t=1}^{n}Rt}\right)\times 100$$

(2) $$f_{2}=50\times \log_{10}\lceil \frac{100}{\sqrt{1+\frac{\sum_{t=1}^{n}{\left(Rt-Tt\right)}^{2}}{n}}}\rceil$$

where *n* is the number of dissolution sampling times, and *Rt* and *Tt* are the mean percent dissolved at each time point, *t*, for the experimental and predicted values of drug released, respectively.

## 3. Results and Discussion 

### 3.1. Printing Process

With 5.0% of the water in screening formulations, printlets were successfully fabricated with an exposure time of 100 s. There was no solidification of the resins with lower exposure time. The increase in water content up to 10.1% required exposure time to be at least 400 s, and with 30.0% water in the formulation, printing was possible at exposure time of 800 s due to which the process lasted for a long time. The water content affected the exposure time to the light projector so that with the increase in the content of water in the formulation, longer exposure time was required, and that was criteria for setting printing parameters in the way presented in Table 2. The minimal exposure time which lead to solidification was selected to keep printing time as short as possible.

It was observed that for every formulation it is necessary to find adequate printing parameters with a trial and error approach because there is no guideline for process parameters selection for mixtures containing photopolymers for pharmaceutical application. A similar observation was reported in the study by Robles-Martinez et al. [24]. Exposure time for every formulation was longer than reported in the published paper by Kadry et al., but in this published paper 2-hydroxy-4′-(2-hydroxyethoxy)-2-methylpropiophenone was used as the photo-initiator, theophylline as the active substance, and the content of the formulation was different as well as their characteristics [11].

### 3.2. Characterization of Printlets

#### 3.2.1. Physical and Mechanical Properties and Drug Content

A DLP printer was able to fabricate 3D printlets with ibuprofen similar to results obtained by Martinez et al [9]. A DLP printlet as well as a 3D model are presented in Figure 2. All fabricated printlets had a smooth surface and consistency in shape. Measured tablet weight, dimensions, hardness, and drug load (mean ± SD) are shown in Table 3.

For better determination of the effects of the formulation factors on obtained mechanical characteristics of printlets, the content of PEGDA, PEG 400, and water were evaluated as the input variables for D-optimal mixture design. Three responses, weight, hardness, and drug load, separately, were fitted to linear, quadratic, special cubic, and full cubic models. The best-fitting mathematical model was selected based on several statistical parameters including adjusted R-squared, predicted R-squared, and predicted residual sum of square (PRESS) (Table 4). The focus was on the model maximizing the adjusted R-squared and the predicted R-squared. The linear model was considered the best fitted model for each of the three responses. 

Mathematically, the relationship for the studied variables was expressed in the following Equations (3)–(5) in actual values. Dimensions of printlets were similar to 3D model but variation in mass and dimension became greater for printlets containing more water. From Equation (3), water had the greatest impact on weight. Water dilutes the formulation, reduces viscosity, and consequently the reproducibility of printing with SLA printer [9]. Instead of the advantages of the DLP printer, previously mentioned, the reproducibility problem with customized resins has not been overcome with this technology.

(3) weight=2.68392×PEGDA+3.78589×PEG400+6.42698×water

(4) hardness=1.52104×PEGDA−0.53285×PEG400−0.28236×water

(5) drug load=0.11399×PEGDA+0.24544×PEG400+0.53252×water

From results and Equation (4) it was observed that the content of PEGDA affected the hardness of the printlets. For printlets with a higher content of PEGDA, greater force was required to break printlets. PEG 400 and water had negative effects. With a higher content of PEG 400 or water, a lower force was measured to break the printlet. Printlet F7 was too elastic, and the hardness tester could not break them. Content of ibuprofen in printlets was greatly affected by the amount of water, with higher water content higher drug content was observed. From Equation (5) there was also a positive effect of PEGDA and PEG 400 on drug load. In a research paper by Martinez et al. it had been demonstrated that the solubility of ibuprofen was increased with the presence of solvents like polyethylene glycol 300, which decreased the polarity of the aqueous solution [9]. Even if the represented mathematical models do not achieve high values of *R*^2^ (*R*^2^ values reached 0.58, 0.57, 0.62, respectively), information extracted through the analysis of the mathematical expressions can help to improve understanding of the effects of formulation factors on characteristics of printlets.

#### 3.2.2. Dissolution Test

**Figure 3:**
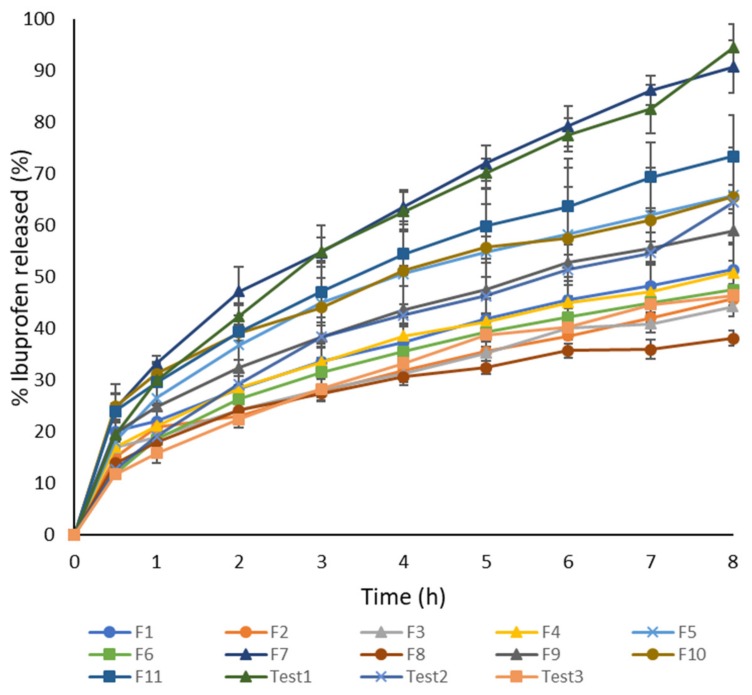
Dissolution profiles of ibuprofen printlets F1–F11 and Test 1–Test 3, Δ exposure time 400 s, × exposure time 500 s, ▪ exposure time 600 s, • exposure time 800 s.

**Figure 4:**
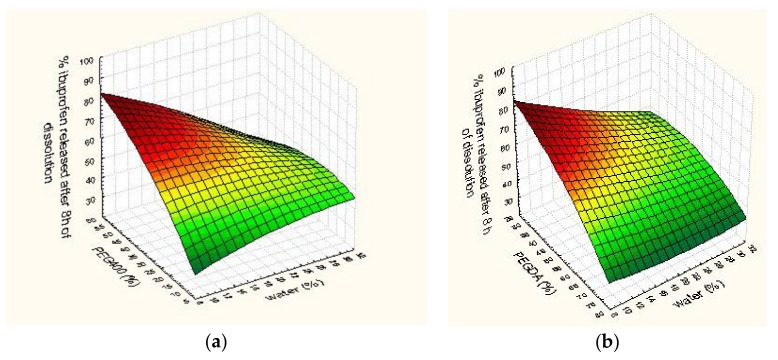

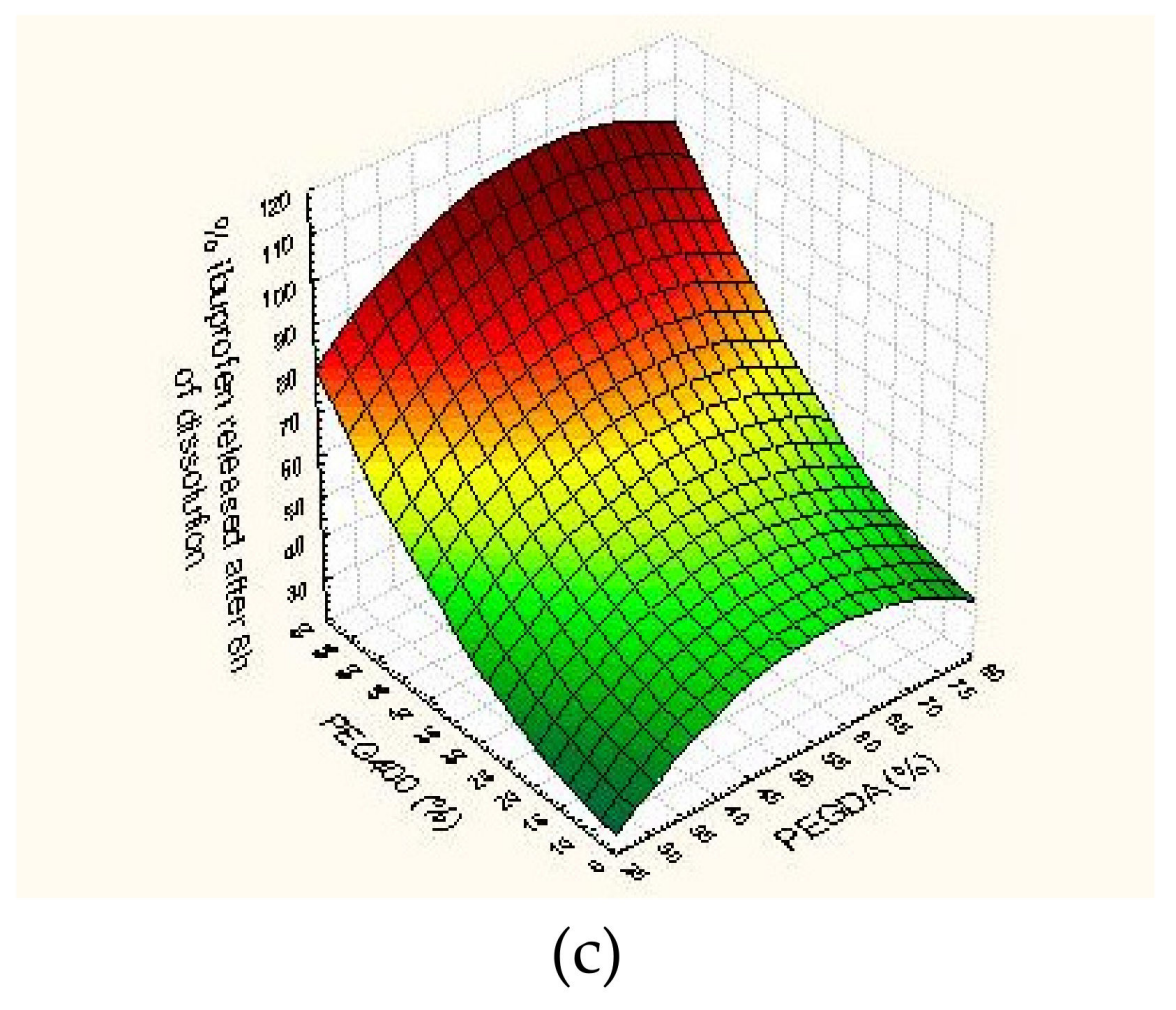
Interaction effect of excipients on ibuprofen released after 8 h of dissolution. (**a**) x—polyethylene glycol (PEG) 400 (%), y—water (%), (**b**) x—polyethylene glycol diacrylate (PEGDA) (%), y—water (%), (**c**) x—PEG 400 (%), y—PEGDA (%), z axis on all graphics—cumulative % of ibuprofen released after 8 h of dissolution test.

Dissolution profiles for all formulations are shown in Figure 3. Printlets fabricated with cross-linkable photoreactive polymers, such as PEGDA, remained intact throughout the dissolution test similar to published studies [11,25]. The fastest dissolution after 8 h was from formulation F7 (90.72 ± 5.06%) that had the highest concentration of PEG 400 (54.6% *w*/*w*), and the slowest dissolution after 8 h was from F8 (38.04 ± 1.41%) that had the lowest concentration of PEG 400 (10% *w*/*w*) and high concentration of PEGDA (58.1% *w*/*w*). In this study, it was observed that PEG 400 had a great influence on the drug release profile, as it was concluded in the study by Wang et al. [7] that changes in the ratio of PEGDA/PEG 300 played an important role in drug release rate. The reduction in the concentration of PEGDA probably increases the drug release rate because of the lower degree of cross-linking in the tablet matrix and increases in the proportion of PEG 400 affected the greater molecular mobility in the tablet core. Formulation F2 had a high concentration of PEG 400 (44.1% *w*/*w*) and the lowest concentration of PEGDA (30% *w*/*w*) but dissolution after 8 h was slower than expected (45.69 ± 0.61%) probably because of the interactions of excipients. The effect of excipients on ibuprofen released after 8 h of dissolution on a 3D surface plot is shown in Figure 4. Martinez at al. [9] showed that dissolution is the slowest from the formulation containing no water and gets faster as the water content is increased, but this clear proportion between water and dissolution rate could not be observed in this study. Martinez et al. used printing with the same process parameters for all formulations, but for the formulations and printer used in this study, it was necessary to adjust the exposure time. By comparing these observations, it can be concluded that not only excipients and their interactions but also printing process parameters could effect drug dissolution rate. Effects of excipients could not be evaluated just by observing modulation in their concentration; it was necessary to apply advanced software. Content of PEGDA, PEG 400, and water were evaluated as the input variables for D-optimal mixture design to determinate their effects on drug release, but the proposed mathematical model was not significant. Because the relationships between the drug release profile of 3D DLP printlets and formulation factors were not well understood, artificial neural networks were used for further research.

#### 3.2.3. Drug Release Kinetic

To interpret the mechanism of drug release from the printlets, data were fitted into various kinetic models such as zero-order, first-order, the Higuchi equation, and the Korsemeyer–Peppas equation. The highest *R*^2^ coefficient determines the suitable mathematical model that best describes drug release kinetics and n gave insights into the mechanism of drug release [26,27]. The most proper model fitted to data based on having the closest *R*^2^ to 1 was the Higuchi model (*R*^2^ was between 0.9746 to 0.9993 for all formulations, Table 5) meaning drug release was afforded through a diffusion process, square root time dependent. In formulations F2 and F the optimum was predominately zero order kinetics but *R*^2^ for the Higuchi model was also high. The values of n less than 0.45 reveal that the diffusion pattern is a kind of Fickian diffusion and values of n between 0.45 and 0.89 reveal that the diffusion pattern is anomalous transport [28]. In the evaluated formulations there was predominately Fickian diffusion as a mechanism of drug release, and during the dissolution test no erosion or swelling of printlets was observed. 

### 3.3. Development of Artificial Neural Network Models

**Figure 5:**
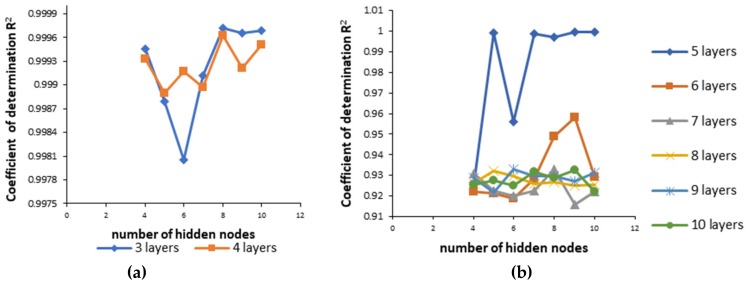
Coefficient of determination (*R*^2^) for neural network 1 with different numbers of hidden nodes and layers (**a**) for 3 and 4 layers (**b**) for 5 to 10 layers.

**Figure 6:**
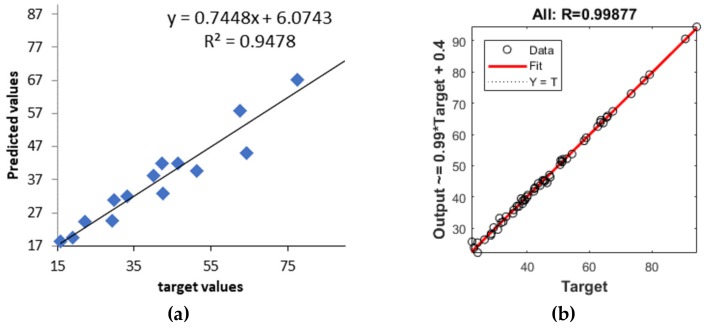
Predicted and experimental cumulative % of ibuprofen release (**a**) for the test dataset in neural network 1 and (**b**) for the whole dataset (training, validation, and test) in neural network 2.

(1) Neural network 1. In the process of creating the most appropriate neural network 1 it was found that increasing the number of layers decreased the coefficient of determination (Figure 5). One hidden layer is normally adequate to provide an accurate prediction and more than one hidden layer can be used for modeling complex problems [29]. Selected MLP had a minimum root mean square (RMS = 0.0296) and the highest coefficient of determination (*R*^2^ = 0.9994) for obtained vs. predicted values of cumulative drug release for two formulations. Hence, a network consisting of three input and five output units, with eight hidden units arranged in a single hidden layer was selected. MLP was tested with a set of test data. Three test formulations (Test 1, 2, 3) were prepared and examined in the same test conditions as formulations F1–F11. A correlation plot was constructed of the experimentally obtained responses and those predicted by MLP. The square coefficient *R*^2^ was 0.9478 (Figure 6a).
(2) Neural network 2. For the second version of the ANN, where exposure times were used as inputs as well as percentage of PEGDA, PEG 400, and water, correlation plots of predicted and obtained values of drug release for all formulations (training, validation, and test) showed that the MLP model had a regression plot with coefficient *R*^2^ = 0.99877, which indicated that the optimum MLP model was reached (Figure 6b). An optimal neural network with neural network 2 was achieved using five hidden layers with the number of units being 5, 5, 6, 5, and 6 per layer. The data set consisted of training (90% of samples) and validation (10% of samples) subsets.

**Figure 7:**
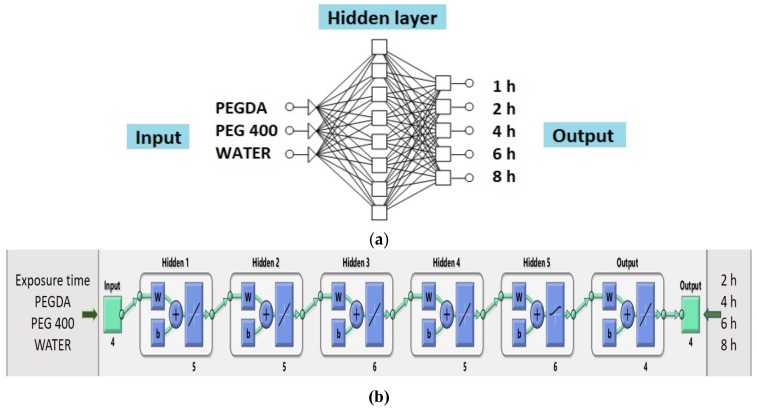
(**a**) Architecture of neural network 1 and (**b**) architecture of neural network 2.

Architecture of developed neural networks is presented in Figure 7.

### 3.4. Optimization and Characterization of Optimal Formulation

**Figure 8:**
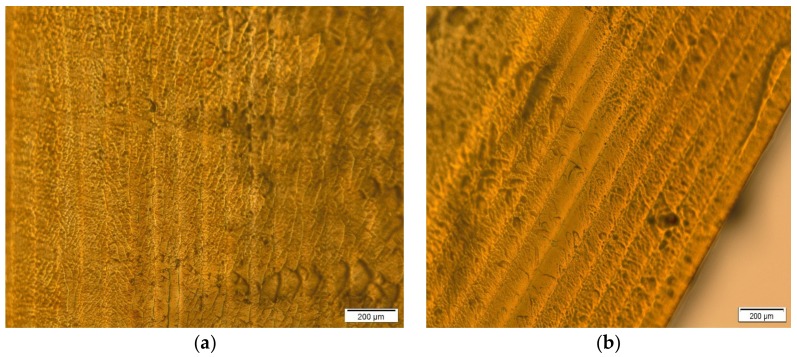
(**a**) Cross-section of placebo tablet; (**b**) cross-section of optimal tablet

The optimal formulation according to the desirability function approach consisted of: PEGDA 30%, PEG 400 52.89%, water 12.02%, riboflavin 0.10%, and ibuprofen 5.00%, and printing was done in the same way as test formulations with exposure time of 400 s, bottom exposure 800 s, layer thickness 0.1, and 10 bottom layers. Predicted drug release at time points of 2, 4, 6, and 8 h was 41.96%, 63.34%, 70.00%, and 79.99% respectively. Fabricated optimal and appropriate placebo formulation was observed under a polarized light microscope and cross-sections are shown in Figure 8. On cross-section of printlets, layers were clearly visible which demonstrated the printing process, but inside of layers in both placebo and optimal printlet undefined structures could be observed. The reasons for their appearance have not been clarified. 

**Figure 9:**
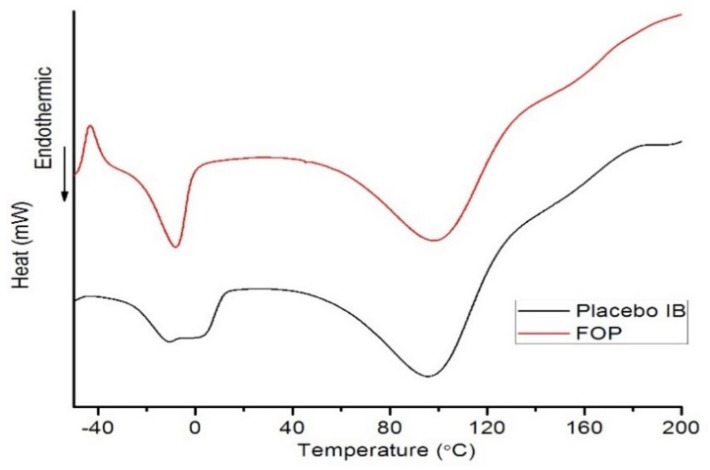
Differential scanning calorimetry (DSC) curves of placebo and optimal printlet.

DSC curves of placebo and optimal printlets are represented in Figure 9. The combination of a sharp peak near 0 °C and a broad peak below 0 °C was observed for the optimal printlet, suggesting co-existence of free and loosely bound water in this formulation. Loosely bound water is associated with non-freezing water and interacts weakly with the ether oxygen, a hydrogen bonded complex between water molecules similar to that in bulk water [30]. The broad endotherm near 100 °C in both placebo and optimal formulations reflects water loss upon heating [9]. No melt endotherm characteristics for ibuprofen are seen, indicating that the drug dissolves in the polymer and/or the water. The solubility of the drug is increased with the presence of solvents like PEG 400, which decreases the polarity of the aqueous solution [9,31]. The exothermic peak near −40°C present in optimal printlets and non-present in placeboes indicates a glass transition temperature of ibuprofen and the presence of ibuprofen in the amorphous phase [32,33]. 

**Figure 10:**
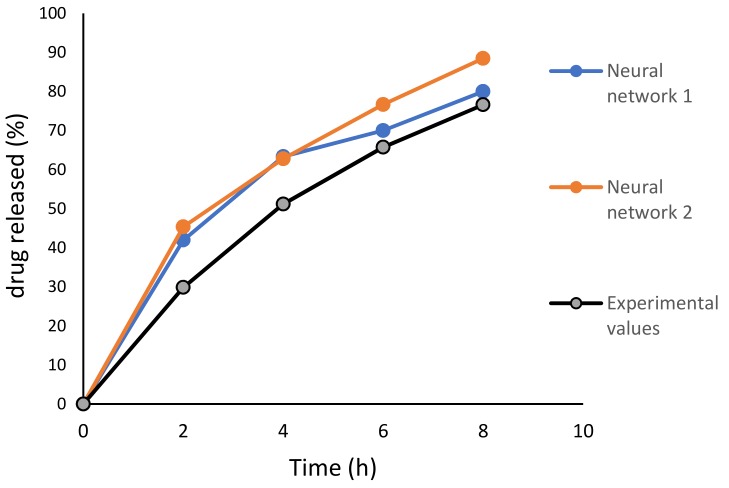
Experimental and predicted dissolution test profiles.

A dissolution test was performed under the same conditions as the test formulations and results are represented in Table 6 and graphically in Figure 10. For the optimal ibuprofen DLP printlet, comparison of release profiles predicted by neural network 1 and neural network 2 and experimental results was done by calculation of *f*_1_ and *f*_2_. Obtained values for neural network 1 are *f*_1_ = 14.30 and *f*_2_ = 52.15, and for neural network 2 are *f*_1_ = 22.34 and *f*_2_ = 44.91.

Two different neural networks were developed to test possibilities of understanding the effect of excipients on ibuprofen release. After comparison of predicted and experimental values of in vitro dissolution at the corresponding time points for optimized formulation, the *R*^2^ experimental vs. predicted value was 0.9811 (neural network 1) and 0.9960 (neural network 2). These values are very close to 1.0, with neural network 2 having a slightly higher *R*^2^ value compared to neural network 1. In machine learning, the correlation coefficient and coefficient of determination are usually adopted as evaluation metrics for regression problems. However, the correlation coefficient and the coefficient of determination cannot properly evaluate the performance of the pharmaceutical formulation prediction models. Thus, specific criteria suitable for pharmaceutics should be introduced to evaluate the model performance [34]. In vitro dissolution profiles can be compared by a model-independent method which includes the difference factor (*f*_1_) and the similarity factor (*f*_2_) [35]. Obtained values of *f*_1_ and *f*_2_ for neural network 1 and 2 showed that neural network 1 gave a similar dissolution profile to obtained experimental results. In developing an optimal formulation, the importance of the first two goals was set with two pluses, and the importance of the next two goals (drug release at 6 and 8 hours) was set with three pluses. From the profiles, it is visible that predicted values at 6 and 8 hours were closer to the real values. Neural network 2 was created with combination of formulation and process parameters. Generally, the main limitation regarding the neural networks is the small number of experiments available, as a higher number of experiments would increase the accuracy of the neural network and this will be done in future studies. ANN with possibilities to provide an understanding of the relationship of input–output variables and give better insights into the effects of excipients and process parameters on dissolution rate could help in optimization of formulating processes and printing printlets according to patient’s needs. 

There are a lot of printing process and formulation parameters and their effects on printlet characteristics are still unknown. Further research will be conducted with the aim to investigate the applicability of combination of ANN and DLP technologies for other drugs and to investigate the effect of formulation and process parameters on characteristics of printlets matrix created with 3D printing technology.

## 4. Conclusions

DLP technology, as a type of 3D printing technology, can be used for the production of extended-release ibuprofen printlets with PEGDA, PEG 400, and water as the main ingredients, and riboflavin as a photo-initiator. It is necessary to adjust printing parameters for every formulation because of the effect of excipients on the success of printing. The relationship between excipients and drug release in tested formulations is complex and non-linear. Artificial neural networks with their ability to generalize can be a useful tool for understanding the effects of excipients on printlets characteristics with the aim to print printlets with the desired drug release. No single software or modeling algorithm can solve “all” problems, but for better prediction and optimization, application of different softwares can be a helpful method. In this study it was demonstrated that adequate ANN is able to understand the input–output relationship in DLP printing of pharmaceutics. 

## Figures and Tables

**Table 1:** Composition (% *w*/*w*) of the initial resins used to print the printlets.

Formulation	PEGDA	PEG 400	Water	riboflavin	ibuprofen
**F1**	32.10	32.60	30.00	0.10	5.00
**F2**	30.00	44.10	20.50	0.10	5.00
**F3**	74.60	10.00	10.10	0.10	5.00
**F4**	62.40	21.80	10.50	0.10	5.00
**F5**	50.60	34.00	10.00	0.10	5.00
**F6**	65.80	11.20	17.70	0.10	5.00
**F7**	30.00	54.60	10.00	0.10	5.00
**F8**	58.10	10.00	26.60	0.10	5.00
**F9**	39.30	45.30	10.00	0.10	5.00
**F10**	46.20	23.10	25.40	0.10	5.00
**F11**	40.40	35.60	18.70	0.10	5.00
**Test 1**	35.00	47.90	12.00	0.10	5.00
**Test 2**	55.00	24.90	15.00	0.10	5.00
**Test 3**	65.00	7.90	22.00	0.10	5.00
**F placebo**	42.50	42.40	15.00	0.10	0.00

**Table 2:** Printing process parameters.

Formulation	Exposure Time (s)	Bottom Exposure (s)	Layer Thickness (mm)	Bottom Layers
F1	800.00	800.00	0.10	10.00
F2	800.00	800.00	0.10	10.00
F3	400.00	800.00	0.10	10.00
F4	400.00	800.00	0.10	10.00
F5	500.00	800.00	0.10	10.00
F6	600.00	800.00	0.10	10.00
F7	400.00	800.00	0.10	10.00
F8	800.00	800.00	0.10	10.00
F9	400.00	800.00	0.10	10.00
F10	800.00	800.00	0.10	10.00
F11	600.00	800.00	0.10	10.00
Test 1	400.00	800.00	0.10	10.00
Test 2	500.00	800.00	0.10	10.00
Test 3	600.00	800.00	0.10	10.00
F placebo	600.00	800.00	0.10	10.00

**Table 3:** Measured tablet weight, dimensions, hardness, and drug load (mean ± SD)**.**

Formulation	Weight (mg)	Diameter (mm)	Thickness (mm)	Hardness (N)	Drug Load (mg)
F1	387.00 ± 45.20	11.13 ± 0.62	3.00 ± 0.00	47.33 ± 3.21	24.11 ± 2.51
F2	378.00 ± 29.00	10.86 ± 0.31	3.09 ± 0.20	32.00 ± 17.00	23.00 ± 1.58
F3	323.40 ± 21.60	10.81 ± 0.31	3.00 ± 0.00	108.33 ± 23.71	15.00 ± 1.00
F4	296.70 ± 4.50	10.17 ± 0.26	3.02 ± 0.04	92.33 ± 29.02	14.40 ± 0.22
F5	354.40 ± 21.10	10.55 ± 0.38	3.00 ± 0.00	33.00 ± 4.58	22.30 ± 0.13
F6	278.90 ± 11.50	10.04 ± 0.09	3.00 ± 0.00	132.33 ± 18.88	18.30 ± 0.75
F7	345.10 ± 32.70	10.52 ± 0.32	2.99 ± 0.02	n.d.^1^	21.70 ± 2.05
F8	400.10 ± 42.90	12.40 ± 0.55	2.97 ± 0.23	29.67 ± 3.51	27.10 ± 2.91
F9	340.50 ± 19.50	10.60 ± 0.17	2.94 ± 0.13	19.00 ± 8.66	23.00 ± 1.13
F10	375.00 ± 28.70	11.53 ± 0.43	2.92 ± 0.11	37.00 ± 16.52	25.80 ± 1.98
F11	377.50 ± 37.30	11.40 ± 0.47	2.99 ± 0.12	35.00 ± 24.25	25.50 ± 2.53

^1^ n.d. not determined

**Table 4:** Model summary statistics.

**Weight**	**Linear**	**Quadratic**	**Special Cubic**	**Cubic**
Adjusted *R*^2^	0.4828	11,760.57	0.0573	0.5331
Predicted *R*^2^	0.2042	−2.6704	−4.744	−15888.43
PRESS	11,760.57	54,239.56	84,882.21	2.35 × 10^8^
**Hardness**	**Linear**	**Quadratic**	**Special Cubic**	**Cubic**
Adjusted *R*^2^	0.4575	0.5454	0.4311	n.d.
Predicted *R*^2^	0.0542	−1.4961	−4.3319	n.d.
PRESS	13,171.03	34,759.87	74,249.53	n.d.
**Drug load**	**Linear**	**Quadratic**	**Special Cubic**	**Cubic**
Adjusted *R*^2^	0.5184	0.6846	0.6145	0.7212
Predicted *R*^2^	0.2228	−0.1716	−0.8126	−9,486.5367
PRESS	139.12	209.72	324.46	1.70 × 10^6^

^1^ n.d. not determined

**Table 5:** Parameters obtained by fitting dissolution data to various mathematical models.

Formulation	Zero Order		First Order		Higuchi		Korsmeyer–Peppas		
	k_0_	*R* ^2^	k_1_	*R* ^2^	k_h_	*R* ^2^	k_kp_	*R* ^2^	*n*
**F1**	0.0707	0.9859	0.0021	0.9428	1.9807	0.9945	5.3769	0.9780	0.3588
**F2**	0.0643	0.9881	0.0022	0.9348	1.7921	0.9861	3.9965	0.9777	0.3843
**F3**	0.0614	0.9866	0.0021	0.9498	1.7126	0.9886	4.5005	0.9739	0.3619
**F4**	0.0727	0.9642	0.0023	0.8935	2.0614	0.9982	4.1796	0.9977	0.4024
**F5**	0.0997	0.9379	0.0025	0.8345	2.8606	0.9922	3.9337	0.9950	0.4609
**F6**	0.0744	0.9427	0.0026	0.8285	2.1292	0.9940	2.3934	0.9932	0.4895
**F7**	0.1445	0.9775	0.0027	0.8961	4.0722	0.9985	4.7498	0.9985	0.4767
**F8**	0.0510	0.9285	0.0020	0.8493	1.4654	0.9871	4.0217	0.9962	0.3671
**F9**	0.0856	0.9746	0.0023	0.9089	2.4164	0.9993	4.8273	0.9972	0.4027
**F10**	0.0857	0.9591	0.0020	0.8963	2.4347	0.9957	7.4583	0.9968	0.3489
**F11**	0.1082	0.9744	0.0023	0.9089	3.0557	0.9989	5.5710	0.9958	0.4147
**Test 1**	0.1552	0.9758	0.0031	0.8732	4.3715	0.9959	3.0129	0.9980	0.5535
**Test 2**	0.1045	0.9641	0.0031	0.8563	2.9500	0.9891	1.8925	0.9944	0.5656
**Test 3**	0.0776	0.9685	0.0029	0.8875	2.1940	0.9959	1.9670	0.9969	0.5144
**F optimal**	0.1286	0.9892	0.0029	0.9516	3.5609	0.9749	3.5776	0.9544	0.4872

k_o_—zero order rate constant, k_1_—first order rate constant, k_h_—Higuchi dissolution constant, k_kp_—Korsmeyer release rate constant, *R*^2^—coefficient of determination, n—drug release exponent,

**Table 6:** Predicted and experimental in vitro release values at time points of 2, 4, 6, and 8 h for optimal formulation of 3D DLP printlets.

Time (h)	Predicted Values (%) Neural Network 1	Predicted Values (%) Neural Network 2	Experimental Values (%)
2	41.96	45.37	29.85
4	63.34	62.77	51.18
6	70.00	76.66	65.73
8	79.99	88.46	76.60

## Supplementary Materials

The following are available online at https://www.mdpi.com/1999-4923/11/10/544/s1: Table S1: Dataset for neural network 1 and Table S2: Dataset for neural network 2.
