# Supplementary material for: Optimization and Prediction of Ibuprofen Release from 3D DLP Printlets Using Artificial Neural Networks
Source: Pharmaceutics. 2019 Oct 18;11(10):544. doi: 10.3390/pharmaceutics11100544 (PMC6835658; doi:10.3390/pharmaceutics11100544)
Supplement: Supplementary file 1 [file pharmaceutics-11-00544-s001.pdf]

pharmaceutics

Supplementary Materials: Optimization and Prediction of Ibuprofen Release from 3D DLP Printlets Using Artificial Neural Networks

Marijana Madzarevic, Djordje Medarevic, Aleksandra Vulovic, Tijana Sustersic, Jelena Djuris, Nenad Filipovic and Svetlana Ibric

Table S1. Dataset for Neural network 1.

Neural network 1								
	Input (composition % *w/w*)			Output (% drug released)				
**Formulation**	**Pegda**	**Peg 400**	**water**	**1 h**	**2 h**	**4 h**	**6 h**	**8 h**
F1	32.100	32.600	30.000	21.910	28.350	37.260	45.461	51.320
F2	30.000	44.100	20.500	20.797	22.998	31.704	38.482	45.696
F3	74.600	10.000	10.100	18.797	24.080	31.128	40.033	44.130
F4	62.400	21.800	10.500	21.011	28.460	38.484	45.010	50.783
F5	50.600	34.000	10.000	26.420	36.715	50.557	58.265	65.765
F6	65.800	11.200	17.700	18.384	26.301	35.470	42.142	47.393
F7	30.000	54.600	10.000	33.091	47.153	63.481	79.254	90.725
F8	58.100	10.000	26.600	17.870	24.112	30.579	35.707	38.042
F9	39.300	45.300	10.000	24.785	32.312	43.570	52.708	58.916
F10	46.200	23.100	25.400	31.116	39.129	51.164	67.483	65.472
F11	40.400	35.600	18.700	29.460	39.270	54.380	63.590	73.350
Test1	35.000	47.900	12.000	29.910	42.310	62.590	77.480	94.370
Test2	55.000	24.900	15.000	18.950	29.230	42.510	51.310	64.410
Test3	65.000	7.900	22.000	15.720	22.300	33.220	40.140	46.360

Table 2. Dataset for Neural network 2.

Neural network 2								
	Input (composition % *w/w* and exposure time (s))				Output (% drug released)			
**Formulation**	**Pegda**	**Peg 400**	**water**	**exposure time**	**2 h**	**4 h**	**6 h**	**8 h**
F1	32.100	32.600	30.000	800.000	28.350	37.260	45.461	51.320
F2	30.000	44.100	20.500	800.000	22.998	31.704	38.482	45.696
F3	74.600	10.000	10.100	400.000	24.080	31.128	40.033	44.130
F4	62.400	21.800	10.500	400.000	28.460	38.484	45.010	50.783
F5	50.600	34.000	10.000	500.000	36.715	50.557	58.265	65.765
F6	65.800	11.200	17.700	600.000	26.301	35.470	42.142	47.393
F7	30.000	54.600	10.000	400.000	47.153	63.481	79.254	90.725
F8	58.100	10.000	26.600	800.000	24.112	30.579	35.707	38.042
F9	39.300	45.300	10.000	400.000	32.312	43.570	52.708	58.916
F10	46.200	23.100	25.400	800.000	39.129	51.164	67.483	65.472
F11	40.400	35.600	18.700	600.000	39.270	54.380	63.590	73.350
Test1	35.000	47.900	12.000	400.000	42.310	62.590	77.480	94.370
Test2	55.000	24.900	15.000	500.000	29.230	42.510	51.310	64.410
Test3	65.000	7.900	22.000	600.000	22.300	33.220	40.140	46.360

© 2019 by the authors. Submitted for possible open access publication under the terms and conditions of the Creative Commons Attribution (CC BY) license (http://creativecommons.org/licenses/by/4.0/).
